# Using artificial intelligence algorithms to predict self-reported problem gambling with account-based player data in an online casino setting

**DOI:** 10.1007/s10899-022-10139-1

**Published:** 2022-07-19

**Authors:** Michael Auer, Mark D. Griffiths

**Affiliations:** 1 neccton GmbH, Davidgasse 5, 7052 Muellendorf, Austria; 2 International Gaming Research Unit, Psychology Department, Nottingham Trent University, 50 Shakespeare Street, NG1 4FQ Nottingham, UK

**Keywords:** Online gambling, Artificial intelligence, Problem gambling, Player tracking, Online casino

## Abstract

In recent years researchers have emphasized the importance of artificial intelligence (AI) algorithms as a tool to detect problem gambling online. AI algorithms require a training dataset to learn the patterns of a prespecified group. Problem gambling screens are one method for the collection of the necessary input data to train AI algorithms. The present study’s main aim was to identify the most significant behavioral patterns which predict self-reported problem gambling. In order to fulfil the aim, the study analyzed data from a sample of real-world online casino players and matched their self-report (subjective) responses concerning problem gambling with the participants’ actual (objective) gambling behavior. More specifically, the authors were given access to the raw data of 1,287 players from a European online gambling casino who answered questions on the Problem Gambling Severity Index (PGSI) between September 2021 and February 2022. Random forest and gradient boost machine algorithms were trained to predict self-reported problem gambling based on the independent variables (e.g., wagering, depositing, gambling frequency). The random forest model predicted self-reported problem gambling better than gradient boost. Moreover, problem gamblers showed a distinct pattern with respect to their gambling based on the player tracking data. More specifically, problem gamblers lost more money per gambling day, lost more money per gambling session, and deposited money more frequently per gambling session. Problem gamblers also tended to deplete their gambling accounts more frequently compared to non-problem gamblers. A subgroup of problem gamblers identified as being at greater harm (based on their response to PGSI items) showed even higher values with respect to the aforementioned gambling behaviors. The study showed that self-reported problem gambling can be predicted by AI algorithms with high accuracy based on player tracking data.

## Introduction

Gambling disorder is a condition which affects around 0.5% of the general adult population (Kessler et al., 2008; Abbott et al., 2018) although there is worldwide variation in problem gambling (PG) prevalence, from below 1% of the adult population, up to around 5-6% (Calado & Griffiths, 2016). Over the past few decades, technology has facilitated gambling, and has led to it being more accessible and available through mobile devices such as tablets and smartphones (Lopez-Gonzalez et al., 2021). Moreover, it has been noted that online gambling is a medium of gambling rather than a type of gambling activity, and that most internet gamblers also gamble offline (Wardle et al., 2011).

Online gambling participation has increased in recent years (Castrén et al., 2018; Chóliz et al., 2021; Gainsbury, 2014; Rodríguez et al., 2017). A recent meta-analysis by Allami et al. (2021) evaluated 57 risk factors from 104 gambling prevalence studies worldwide (with sample sizes ranging from 5327 to 273,946 in the studies examined). The risk factors in the studies were ranked in regard to their association with problem gambling. The risk factor with the highest odds ratio was online gambling. They also reported that continuous forms of gambling (such as slot machines and casino games) were most associated with problem gambling. There are also features of the internet that provide reasons as to why users can spend so long online including the perceived anonymity, affordability, easy accessibility, interactivity, immersion/ dissociation, convenience, and disinhibition facilitation (Griffiths, 2003).

Most reviews of online gambling suggest it is a more ‘dangerous’ or ‘harmful’ medium than offline gambling (e.g., Kuss & Griffiths 2012; Mora-Salgueiro et al., 2021). For instance, Sirola et al. (2018) assessed problem gambling among a sample of 1200 Finnish internet users with the South Oaks Gambling Screen. The results showed that over half of participants who had visited gambling-related online communities were either at-risk gamblers or probable pathological gamblers (54.33%). In three different regression models, visiting gambling-related online communities was a significant predictor for excessive gambling. However, other studies have not found online gambling to be related to increased problem gambling. For instance, Philander and MacKay (2014) used secondary data and found that past-year participation in online gambling was related to a decrease in problem gambling severity, which is the opposite of the popular view in extant literature. Moreover, in one of the few studies that compared offline-only gamblers, online-only gamblers, and mixed-mode gamblers (i.e., those who gambled both online and offline) using a nationally representative sample of British gamblers, Wardle et al. (2011) reported no problem gambling among those who only gambled online. Problem gambling was highest among mixed-mode gamblers followed by offline-only gamblers. The results suggest that the medium of online gambling is not harmful itself but that to those who are vulnerable (e.g., problem gamblers), the online medium could provide heightened risk because of its 24/7 capability.

### Artificial intelligence, behavioral tracking, and gambling markers of harm

The terms ‘artificial intelligence’ (AI), ‘machine learning’ and ‘data science’ are often used interchangeably. However, machine learning refers to a group of advanced statistical methods, whereas AI can be regarded as the outcome of an advanced algorithm (Petit et al., 2021). Online gambling facilitates the application of advanced analytical methods because each and every transaction is assigned to one account and recorded. Auer and Griffiths (2013) argued that good tools which track a player’s behavior should be able to support informed player choice, and also help online gambling operators gain more insight into their players’ behavioral patterns. AI methods have been applied for numerous purposes in gambling research. Several studies have used AI methods to predict voluntary self-exclusion (i.e., Dragicevic et al., 2015; Finkenwirth et al., 2021; Haeusler, 2016; Percy et al., 2016). Two studies have used AI methods to predict self-reported problem gambling (Luquiens et al., 2016; Louderback et al., 2021). Auer and Griffiths (2019) applied AI methods to predict voluntary limit setting among a sample of Norwegian online players. Cerasa et al. (2018) used AI methods to predict personality traits predictive of self-reported problem gambling in a sample of 40 psychiatric patients, recruited from specialized gambling clinics.

One of the innovations in gambling research over the past 15 years is the increasing use of high-quality account-based behavioral tracking data provided by the gambling industry to academic researchers. Both researchers and the gambling industry have utilized player tracking data as a way to try to identify problem gambling (Auer & Griffiths, 2013; Deng et al., 2019). For instance, AI methods were used by Ukhov et al. (2021) to compare online casino players (n = 5000) and online sports bettors (n = 5000) and to see which features were more predictive of problem gambling. The problem gambling sample was large (n = 5000, comprising 2500 online casino players and 2500 online sports bettors, all of who had self-excluded specifically because they had problem gambling issues). They reported that the number of daily wagers and the use of mobile devices (e.g., smartphones) were two of the key predictors of problem gambling for online sports bettors whereas session durations, volume of approved deposits, and use of desktop computers were the key predictors for online casino players. The study concluded that online problem gambling is not homogeneous and that there are behavioral differences in between problem gamblers based on preferred game type.

As a result of a number of meetings between five major gambling operators – *888 Holdings, GVC Holdings* (now called *Entain*), *Sky Betting & Gaming, William Hill*, and *Paddy Power* – the Senet Group developed a set of nine markers of harm to identify problematic gambling (McAuliffe et al., 2022). The Senet Group is an organization which was established in 2014 by the leading high-street bookmakers in the UK which was then taken over by the Betting and Gaming Council (Narayan, 2020). Each of the nine markers (e.g., increase in frequency of gambling, increased deposit frequency, failed deposits, late-night gambling, etc.) is assigned four values (0 = no-risk, 1 = low-risk, 2 = medium-risk, and 3 = high-risk) and the overall score across all nine markers can range between 0 and 27. The overall score is also classified into categories (no-risk = 0–7, Level 1 = 8–9, Level 2 = 10–14, and Level 3 = 15–27) results in a type of intervention (PwC and Responsible Gambling Council, 2017). The markers of harm identify changes in gambling (e.g., yesterday’s deposit was 2.5 times larger than the average deposit for the past six months) as well as an assessment of overall gambling behavior that might be viewed as risky (e.g., making 20 or more deposits in the last 28 days).

In the peer-reviewed literature, McAuliffe et al. (2022) used two datasets from *bwin’s* online sportsbook (one covering 2005–2007, and another covering 2015–2017) to evaluate the prevalence of gambling markers of harm, as well as their intercorrelations, inter-individual and intraindividual stability, and correlations with extreme betting activity, demographic variables, and gambling harm proxies. The authors found that on an average day, less than 1% of players had risk scores high enough to trigger an intervention. They also found that male gender and younger age were not positively correlated with the risk score. They also reported that there were strong associations between the highest risk score during the study period and being a top 6% or top 1% user in terms of number of bets or money wagered.

In the most recent (fifth) edition of the *Diagnostic and Statistical Manual of Mental Disorders* (DSM-5), gambling disorder was identified as a behavioral addiction (American Psychiatric Association, 2013; Catania and Griffiths, 2021a) suggested ways that the DSM-5 criteria could be operationalized using behavioral tracking data. For instance, gambling preoccupation was operationalized in four different ways including the number of hours players spent on the website and the number of wagers and tolerance was operationalized in two different ways including the increase in the number of money deposits over time. They used a sample of 982 online gamblers and the first three months of their gambling activity and concluded that some DSM-5 criteria could be operationalized with player tracking data. Through cluster analysis they identified four types of online gambler (non-problem gamblers, at-risk gamblers, financially vulnerable gamblers, and emotionally vulnerable gamblers), the latter two groups being problem gamblers and accounting for 1.23% of the sample.

### Problem gambling indicators using data from gamblers who have voluntarily self-excluded

A number of studies have examined the profile of gamblers who have utilized voluntary self-exclusion (VSE) tools. Using behavioral tracking data (i.e., the first month of gambling data among players who engaged in VSE because of gambling-related problems), Braverman et al. (2012) reported that the characteristics of first-month betting were an increase in wagering, frequent intensive betting, and high variability in amount of money wagered.

Finkenwirth et al. (2021) compared 2,157 Canadian online gamblers who had requested VSE with 17,526 players who had not voluntarily self-excluded using 20 input variables of gambling behavior. They applied AI algorithms to identify patterns indicative of future self-exclusion. The variance in money bet per session was the most predictive explanatory variable for VSE. Other significant variables were the number of bets, the number of games per session, money bet from promotional offers, amount of money won per day, and the number of sessions per day. Using a different methodology, Haeusler (2016) used payment data from a sample of 2696 *bwin.com* players to predict voluntary self-exclusion utilizing AI algorithms. The study found that the frequency of deposits and the amount of money deposited, the variance of the single amounts withdrawn, the amount of funds subject to reversed withdrawals (when a player initiates a withdrawal of money after winning money on the website and then decides not to and cancels the process), and the use of smartphones to deposit money into their gambling account were found to be positively associated with gambling self-exclusion.

Dragicevic et al. (2015) compared player tracking data from a sample of 347 players who self-excluded with a control sample of 871 players who did not self-exclude. They also compared the efficiency of different AI methods. Their main finding was that self-excluders lost more money than the control group. Their analysis also found that self-excluders made riskier bets than the control group. Catania and Griffiths (2021b) compared players who closed their account due to a specific self-reported gambling addiction with players who chose a six-month account closure option. Players who chose to close their account for six months had low gambling activity and had only registered recently (i.e., just over 50% of gamblers self-excluded within seven days of opening a gambling account, with one-fifth self-excluding within 24 h of opening an account). Catania and Griffiths concluded that players who excluded voluntarily were too different to be treated as a homogenous group and that self-exclusion alone was not a good proxy for problem gambling. Using a variety of machine learning techniques, Percy et al. (2016) reported that the most accurate method in identifying VSE was the random forest method.

Auer and Griffiths (2016) also argued that voluntary self-exclusion should not be used as a proxy measure for problem gambling. They noted that there was no evidence of a direct relationship between long-term self-exclusion and problem gambling and that gamblers self-exclude for various reasons. Moreover, they noted that many problem gamblers never self-exclude and many self-excluders do not have gambling problems and do not exclude for reasons concerning problem gambling.

### Self-reported problem gambling

There are over 20 screens that can assess problem gambling (Stinchfield, 2014). Among the most popular instruments are the South Oaks Gambling Screen (SOGS) and the Problem Gambling Severity Index (PGSI). The SOGS is a 20-item scale and can reliably identify individuals who are likely problem gamblers Duvarci & Varan, 2001; Lesieur & Blume, 1987; Shaffer et al., 1999; Stinchfield 2002). Strong et al. (2003) asserted that the SOGS does not include less severe behavioral items and therefore may not do so well in identifying people who are in the process of becoming problem gamblers.

The Problem Gambling Severity Index (PGSI; Ferris & Wynne 2001) comprises nine items, four of which assess problem gambling behaviors and five that assess negative consequences of gambling. In a sample of 12,299 Canadian adults, Holtgraves (2009) found that one underlying factor explains the nine PGSI questions. Holtgraves (2009) argued that the PGSI presents a viable alternative to the SOGS for assessing degrees of problem gambling severity in a non-clinical context. The PGSI was developed to reflect more socially oriented (rather than clinical) PG aspects (Petry, 2016). To date, the PGSI is arguably the most widely used PG-screening tool currently (Calado & Griffiths, 2016).

Only a couple of studies have reported the association between self-reported problem gambling and player tracking data among the same sample of online players (i.e., Luquiens et al., 2016; Louderback et al., 2021). Luquiens et al. (2016) carried out a survey among online poker players (n = 14,261) which included the Problem Gambling Severity Index (PGSI). Their responses on the PGSI were compared with the tracking data of their actual gambling. Almost one-fifth of the participants who completed the PGSI were classed as problem gamblers (18%). The key risk factors reported for problem gambling were: being male, being aged below 28 years, having 60 + wagering sessions during the one-month study period, losing more than €45 during the one-month study period, depositing 3 + times during a 12-hour period, staking more than €298 during the one-month study period, having more than €1.7 mean loss per session during the one-month study period, and engaging in multi-tabling (playing simultaneously on multiple poker tables).

Louderback et al. (2021) used the Brief Biosocial Gambling Screen (BBGS) to assess self-reported problem gambling among a sample of online gamblers. Their aim was to identify thresholds for low-risk gambling. Among other variables, they measured duration of gambling activity, gambling variability, net loss, amount of money wagered, and changes in gambling behavior as predictive variables. The area under the curve (AUC) in the prediction of the BBGS status was between 0.58 and 0.657. They concluded that wagering €167.97 or less each month, spending 6.71% or less of individual’s annual income on online gambling wagers, losing €26.11 or less on online gambling per month, and demonstrating variability (i.e., standard deviation) in daily amount wagered of €35.14 or less were indicative of low-risk gambling.

Previous papers have claimed that chasing losses can easily be observed by gambling operators or researchers using account-based behavioral tracking data (e.g., Delfabbro et al., 2012; Griffiths & Whitty, 2010). More recently, Challet-Bouju et al. (2020) and Perrot et al. (2018) operationalized chasing losses as either three or more deposits within a 12-hour period or a deposit less than one hour after a previous bet. Both studies clustered large samples of online lottery and sports players and found that frequent session deposits were correlated with high gambling intensity.

### The present study

Gambling regulations in a number of European countries (e.g., UK, Spain, Germany, Sweden, Denmark) require license holders to identify problem gambling and regularly report the number of problem gamblers to regulators. However, there is little research into the actual playing behavior of problematic online gamblers. Luquiens et al.’s (2016) study was based on online poker players and Louderback et al.’s (2021) study was based on relatively old data from 2005 to 2010. Since then, internet gambling – as well as mobile gambling – has significantly increased (McGee, 2020).

The present study utilized a recent sample of European online casino players and analyzed the association between self-reported problem gambling and player tracking data. To the best of the authors’ knowledge, Europe is the most highly regulated online gambling environment which also includes the strictest player protection regulations. For that reason, the authors examined a sample of European online casino players for the present study. Moreover, the authors believe that the present study makes an important academic contribution. The findings will be very helpful for online gambling operators as well as for regulators and policymakers.

There were no specific hypotheses regarding the association between gambling behavior and self-reported problem gambling. However, the study’s main aim was to identify the most significant behavioral patterns which predict self-reported problem gambling. In order to fulfil the aim, the present study analyzed data from a sample of real-world online casino players and matched their self-report (subjective) responses concerning problem gambling with the participants’ actual (objective) gambling behavior. The authors aimed to replicate as many behavioral metrics used in previous research as possible for reasons of comparability. Therefore, the study was necessarily explorative in nature.

## Method

The authors were given access by a European online casino to raw data of all players who had answered the nine questions of the Problem Gambling Severity Index (PGSI) between September 2021 and February 2022. Furthermore, only players who placed at least one wager in the 30 days prior to answering the PGSI items were included in the sample. Players were not actively prompted to answer the PGSI. They could answer the PGSI at any time as it was always available on the website in the gambling operator’s ‘Responsible Gaming’ section. Only the most recent set of answers were used for players who had answered the PGSI multiple times during the study period. The nine PGSI questions are listed in the Appendix 1.

The data comprised each wager and each win as well as each deposit and each withdrawal by all the individuals who met the inclusion criterion (i.e., gamblers who placed at least one wager in the 30 days prior to answering the PGSI). The data also contained the amount of money in the gambling account (balance) before and after each transaction. The authors were also given access to each player’s age and gender. The authors computed gambling sessions based on the raw data. Sessions were computed based on the timestamp of the single wagers. If two wagers were placed within 15 min of each other, the time between those two events counted as gambling session time as has been used in other tracking studies (Hopfgartner et al., 2021). If there was more than 15 min between two wagers, the time between the two events was not counted as belonging to the same gambling session.

### Statistical analysis

For each of the nine PGSI items, players could choose between the categories ‘Never’ (0), ‘Sometimes’ (1), ‘Most of the time’ (2) and ‘Almost always’ (3). Scores ranged between 0 and 27. The authors also had access to the number of seconds between the first click on the PGSI site and the click on the submit button after answering all nine questions. Appendix 2 reports the player tracking features which were computed for each player for the 30 days prior to answering the nine PGSI questions. The player tracking features measure the total number of deposits and bets in the 30 days prior to answering the PGSI as well as average amounts of money wagered per gambling day and per session. Furthermore, the authors had access to data concerning prior self-exclusions (play breaks) as well as voluntary limit-setting data. Two of the player tracking features in the present study were attempts to operationalize and measure chasing losses (i.e., regular gambling account depletion and frequent session depositing). These are operationally defined below.

- *Regular gambling account depletion (i.e., percentage of sessions ending with low account balance)*: The authors had access to the amount of money in the gambling account before and after each wagering transaction (also referred to as the balance). The amount of money in the gambling account after the last game of a session was computed. For each player, the authors computed the percentage of sessions when there was less than €5 in the gambling account at the end of the session. The present authors believe that players who regularly deplete their gambling account may be an indication of chasing and not being able to stop gambling.
- Frequent session depositing (i.e., average number of deposits per session/gambling day): For each player, the authors computed the average number of monetary deposits per session. Depositing frequently in a session may be an indication of chasing after losses and not being able to stop or control gambling (Challet-Bouju et al., 2020).

The authors also applied two widely used artificial intelligence (AI) algorithms to prediction of self-reported problem gambling.

- *Gradient boost machine learning (GBML)*: GBML is a method which fits the data with numerous models that are then aggregated to a final model (Friedman, 2001). GBML can detect linear as well as non-linear patterns.
- *Random forest (RF)*: RF is a popular machine learning method which fits the data with numerous decision trees which are then aggregated into a final model (Liaw & Wiener, 2002). RF can detect linear as well as non-linear patterns. The AI model’s predictive quality was measured using the area under the curve (AUC). A value of 0.5 indicates a low model quality and a value of 1 indicates a perfect fit between the predicted and actual values. Ling et al. (2003) have argued that AUC is a better way to measure the predictive quality of AI models than the percentage of correctly classified records. The AUC is a goodness of fit statistic which can be used to evaluate model quality (Bradley, 1997).

The dependent variable was self-reported problem gambling and the independent variables were player tracking features (listed in Appendix 2). The independent variables reflected the behavior for the 30 days prior to answering the PGSI. In order to find the best fitting configuration, an automatic parameter search was conducted for both (i.e., the random forest and the gradient boost machine algorithms). The optimal parameters were used to compute a random forest and a gradient boost machine algorithm.

AI methods such as the ones chosen in the present study provide little insight into the importance of single variables as predictors of self-reported problem gambling. In order to gain more understanding as to which variable contributed to increased or decreased likelihood of self-reported problem gambling, the authors applied a cluster analysis. Cluster analysis is also referred to as unsupervised learning as it aims to classify data into subgroups (Jain et al., 2008). The algorithm assigns the sample to groups where members of one group are as similar as possible and members of different groups are as dissimilar as possible. In the present study, cluster analysis is simply used as an approach to further understand the relationship between the behavioral metrics and self-reported problem gambling.

The authors used the programming language *Python* (Van Rossum, 2007) to analyze the dataset. The scikit library (Pedregosa et al., 2011) was used for the machine learning algorithms. The models’ performances were visually evaluated via their respective receiver operating characteristic (ROC) curves (Hanley & McNeil, 1982) and numerically via the area under the curve (Bradley, 1997). In order to test the validity of the machine learning models the data were split in to a training and a test set. More specifically, 80% of the data were used to train the models and 20% of the data were used to test the validity of the models.

### Data cleaning and participants

**Fig. 1:**
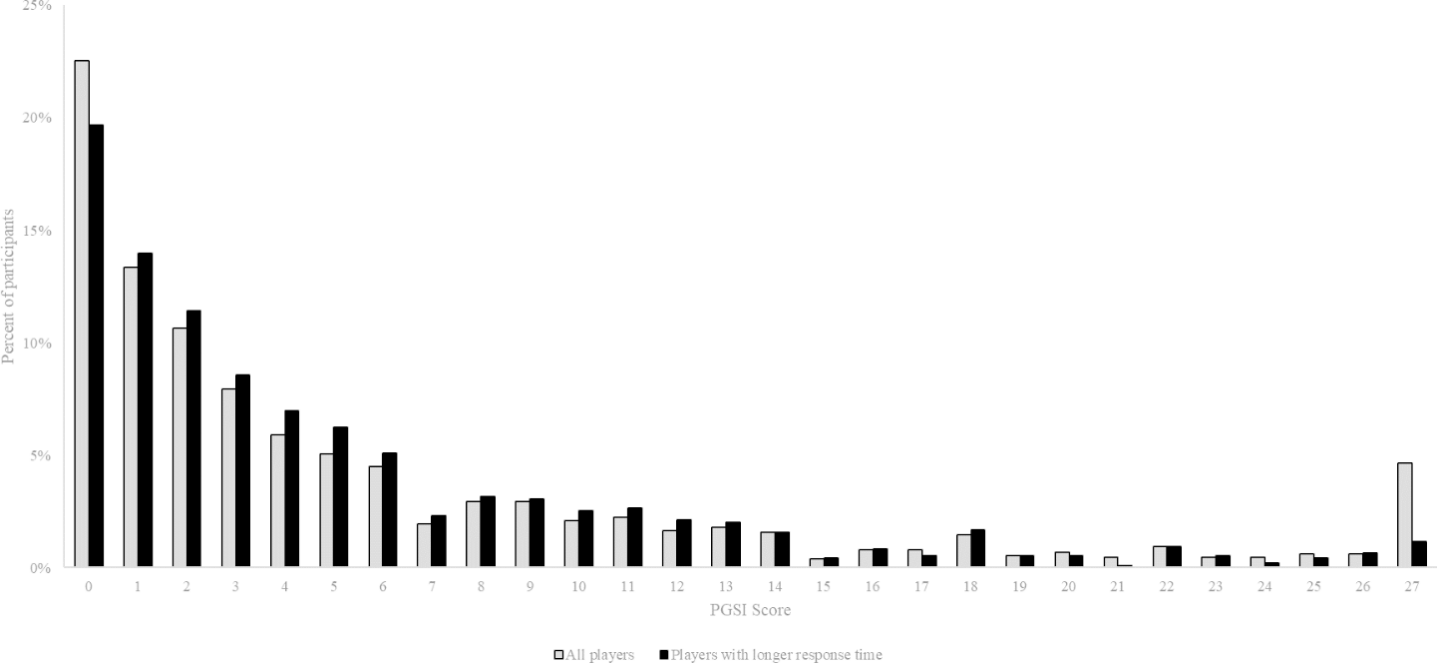
Percentage of players for each PGSI score before and after removing players with short response time

A total of 1,287 players answered the nine PGSI questions between September 2021 and February 2022. This was the time period for which data were made available to the authors. Out of the 1,287 players, 60 players answered all nine questions with *“almost always”* which results in a score of 27 (4.66%). However, only eight players (0.62%) received a PGSI score of 26. The relatively large number of players scoring 27 could be a result of rushing through the nine questions without reading them sufficiently. For that reason, the authors removed players with a very short response time from the data sample. Consequently, 945 players with a reasonable response time were retained. The distributions of the 945 players PGSI scores as well as the original 1,287 players PGSI scores are displayed in Fig. 1. Out of the 945 players, only 11 players had a PGSI score of 27 (1.2%). The data cleaning process also reduced the percentage of players who answered all nine questions with *“never”* from 22.5 to 19.7%. Answering all nine questions with “never” could also have been more likely among the players who rushed through the nine questions without reading them sufficiently. The average age of the 945 players was 41 years (SD = 11.81) and the sample comprised 433 females (46%) and 512 males (54%).

## Results

**Fig. 2:**
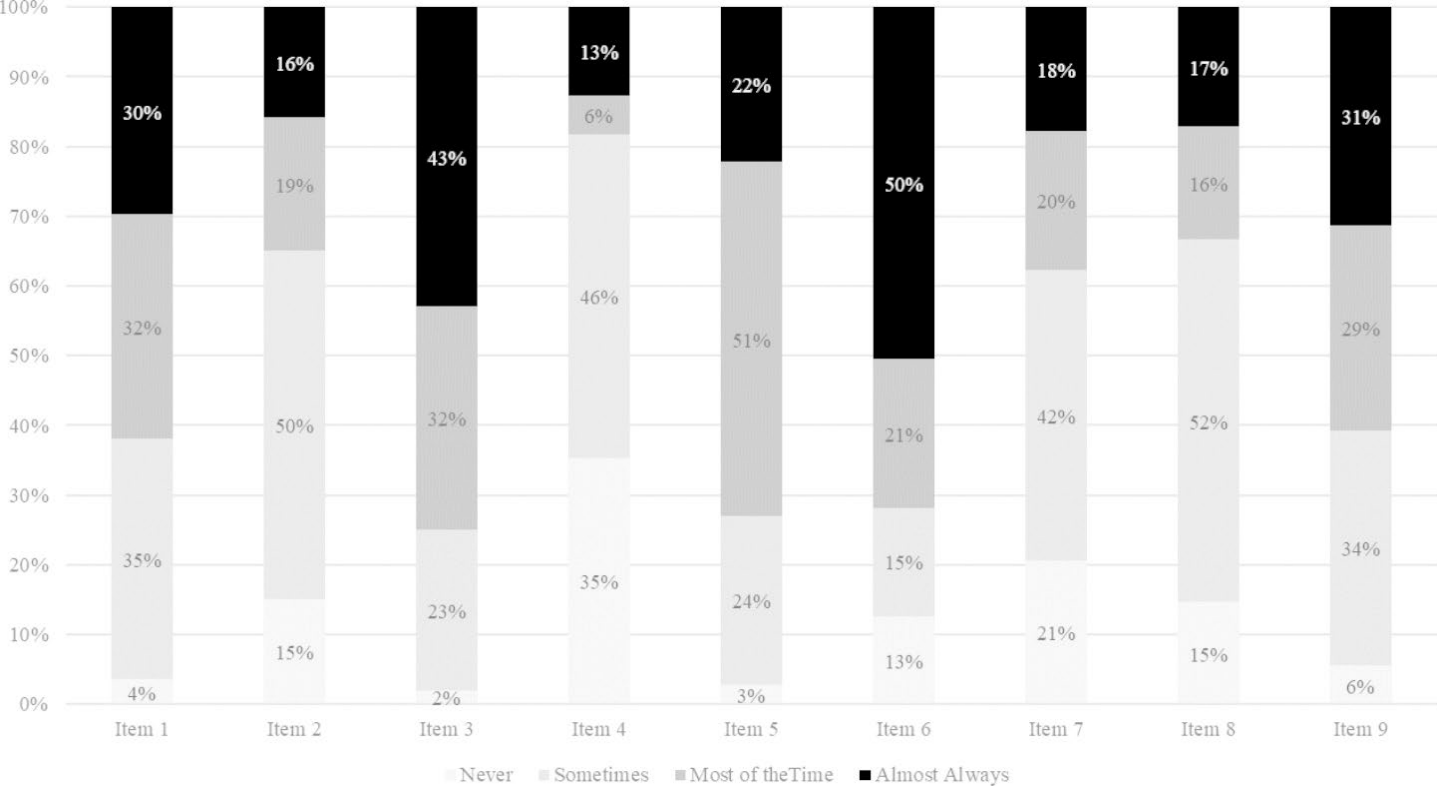
Percentage of players answering each of the nine PGSI items *“never”, “sometimes”, “most of the time”*, and *“almost always”*

Out of the 945 players, 248 players had a PGSI score of 8 or above (26%). A PGSI score of 8 or above indicates probable problem gambling. Figure 2 displays the distribution of the four answers for each of the nine items for the group of problem gamblers. Item 6 (*“Have you felt that gambling has caused you any health problems, including stress or anxiety”*) was answered most frequently (50%) with *“almost always”.* Item 4 (*“Have you borrowed money or sold anything to get money to gamble?”*) was answered least frequently with “almost always” (13%). Item 4 also has the largest percentage of problem gamblers who answered “never” (35%).

### Artificial intelligence models

**Fig. 3:**
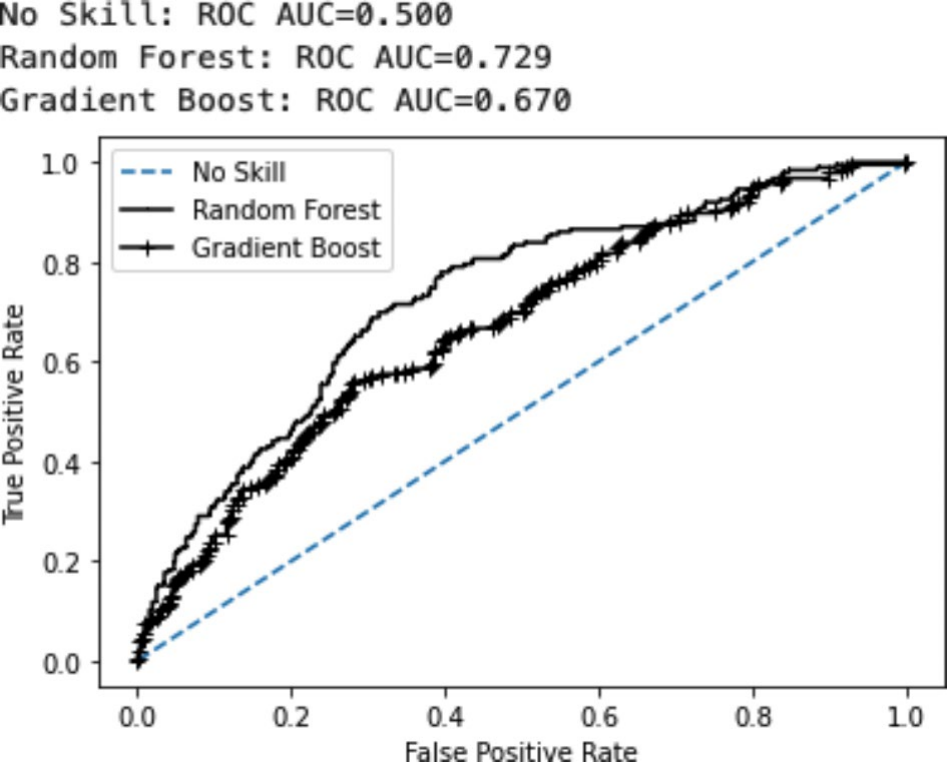
Receiver operating curve of the random forest and a gradient boost algorithm on the test data

Random forest and gradient boost machine algorithms were trained to predict self-reported problem gambling based on the independent variables (e.g., wagering, depositing, gambling frequency). In order to find the best fitting configuration, an automatic parameter search was conducted for both (i.e., the random forest and the gradient boost machine algorithms). The optimal parameters were used to compute a random forest and a gradient boost machine algorithm. Figure 3 reports the Receiver Operating Curve (ROC) as well as the area under the Curve (AUC) values for both algorithms. The ROC reports the percentage of correctly classified problem players (true positive rate/sensitivity) in relation to the percentage of wrongly classified non-problem gamblers (false positive rate/1-specificity; see Narkhede 2018) for different cut-off values of the predicted probability of being a problem gambler.

The area under the ROC is referred to as the area under the curve (AUC). The entire chart is a square. Each side of the square has a length of one which leads to an area of 1 (1 × 1). The area on each side of the diagonal line is 0.5. The diagonal line represents a random model which has an AUC of 0.5. A perfect model which classifies each problem gambler and each non-problem gambler correctly would have an AUC of 1. The larger the AUC the better the model quality. The random forest model’s AUC value computed on the test data was 0.729. This was larger than the gradient boost model’s AUC which was 0.67. This indicates that the random forest model predicts self-reported problem gambling better. The random forest algorithm also reports the most important variables in the model. These were age, amount of money deposited, amount of money bet, number of gambling days, average monetary loss per gambling day, average monetary loss per session, average number of monetary deposits per session, account depletion, and number of play breaks.

### Cluster analysis

**Fig. 4:**
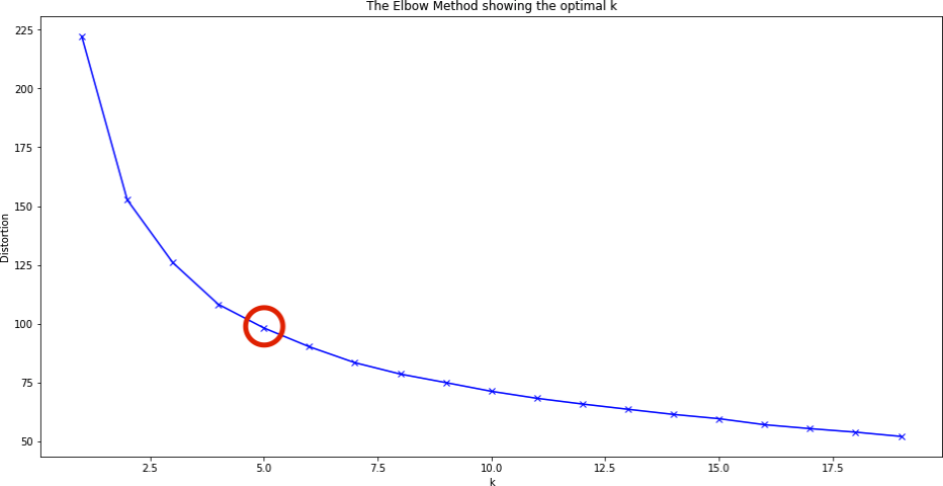
Elbow chart visualizing the optimal number of clusters for the given dataset. (The red circle indicates that four clusters are the best possible solution)

The random forest machine learning algorithm does not report whether there are positive or negative correlations between the explanatory variables and self-reported problem gambling. In order to gain further insight into the behavior of problem gamblers (PGs) compared to non-problem gamblers (NPGs), the authors performed a k-means cluster analysis. The aim was to find clusters of players with a higher percentage of self-reported problem gambling. The previously reported variables with the highest importance in the random forest machine learning algorithm were used in the cluster analysis. A z-score transformation was applied to the variables (Mohamad et al., 2013). After this standardization, each variable carried the same weight in the clustering process. The number of clusters was determined using the elbow method (Kaufmann & Rousseeuw, 1990). The elbow method is a visual approach which displays the within-sum of squares for different numbers of clusters. The optimal number of clusters appears at the so-called elbow where the slope changes most significantly. Figure 4 indicates that a five-cluster solution fitted the data best. The five-cluster datapoint is also indicated by the red circle in Fig. 4. Although Fig. 4 looks similar to Fig. 3, the two are completely unrelated. Figure 4 shows the within-sum of squares for different numbers of clusters and the area under the curve is meaningless for these data.

Table 1 reports the average values for each of the five clusters. Self-reported problem gambling and being female were not used in the cluster analysis. In total, 26% of the 945 players reported problem gambling based on their PGSI responses. The percentage of self-reported problem gambling was different across the clusters. The largest percentage of PGs was found in Cluster 1 (n = 124; 43%). None of the four other clusters had a percentage of PGs above average. Cluster 5 had the lowest percentage of PGs (n = 13; 10%). With a mean age of 31 years, players in Cluster 1 had the lowest mean age. The mean age across all 945 players was 40 years. The percentage of women in Cluster 1 (37%) was also lower than the average percentage of women (45%). Only Cluster 3 had a lower percentage of women (33%). On average, players in Cluster 1 deposited €385 (in the 30 days prior to answering the PGSI) which was lower than the total average of €568. Only players in Custer 3 deposited less money (€269). On average, players in Cluster 1 bet €2724 which was lower than the total average of €5922. Only players in Cluster 3 deposited less money (€2372).

On average, players in Cluster 1 gambled on 5.53 days during the previous 30-day period which was less frequently than the total average of seven days. Only players in Cluster 3 gambled less frequently (5.32 days). Players in Cluster 1 lost €45.77 per gambling day during the previous 30-day period which was more than the total average loss per gambling day (€15.24). Only players in Cluster 3 lost more money per gambling day (€58.14). A negative loss metric refers to a loss which means the amount bet was larger than the amount won. A positive loss metric refers to a win. On average, players in Cluster 4 won €91.70 per gambling day. Players in Cluster 1 deposited 1.37 times per session. This was the highest value across all clusters. In total, players deposited 1.11 times per session. Players in Cluster 1 lost €33.14 per session which was higher than the average loss across all players (€-11.31). Only players in Cluster 3 lost more per session (€33.50). Moreover, 93% of players in Cluster 1 usually gambled until they had less than €5 on their gambling account. In total, this behavior occurred among 67% of all players. All other clusters respective values were lower. In total, 21% of players took a play break. Cluster 1 had the largest percentage of players taking play breaks (27%).

**Table 1:** Average values for each of the five computed clusters (with clusters sorted according to size)

	Cluster 1	Cluster 2	Cluster 3	Cluster 4	Cluster 5	Total
PG	43%	25%	23%	13%	10%	26%
Age	31	35	54	45	45	40
Female	37%	43%	33%	79%	47%	45%
Amount of money deposited (€)	385	630	269	470	1 361	568
Amount of money bet (€)	2 724	6 897	2 372	8 340	13 339	5 922
Number of gambling days	5.53	6.00	4.91	5.32	19.71	7
Average monetary loss per gambling day (€)	- 45.77	- 18.85	- 58.14	91.70	- 1.73	- 15.24
Average number of deposits per session	1.37	1.13	1.14	0.77	0.85	1.11
Average monetary loss per session (€)	- 33.14	- 6.26	- 33.50	43.31	- 1.49	- 11.31
Percentage of sessions ending with low account balance	93%	54%	87%	15%	59%	67%
Play break (yes/no)	27%	20%	24%	14%	14%	21%
Number	287	209	176	141	132	945
Percentage	30%	22%	19%	15%	14%	

### Greater harm problem gamblers

Next, the authors selected a subgroup of PGs based on specific PGSI items. The nine items in the PGSI are equally weighted but some items are far more indicative of problem gambling than others. For instance, responding with the answer ‘almost always’ to some items on the PGSI (e.g., *“Have you felt that you might have a problem with gambling?”, “Has gambling caused you any health problems, including stress or anxiety?”* and *“Has your gambling caused any financial problems for you or your household?”*) are much more strongly associated with problem gambling than items like borrowing money from others to gamble and being criticized by others for gambling. Out of the 248 players which scored at least eight or above on the PGSI, 79 players answered at least one of the three aforementioned questions to be more indicative of gambling harm with *“almost always”.* Moreover, 8.4% of the 945 players were in the subgroup of PGs.

Table 2 reports average values of the 248 PGs, the subgroup of 79 greater harm problem gamblers (GHPGs) and the remaining 697 NPGs. The three numbers do not sum up to the sample size, because the 79 GHPGs are included in the 284 PGs. PGs as well as the GHPGs were younger than NPGs. PGs were on average 37 years old, the GHPGs were on average 36 years old, and NPGs were on average 43 years old. A total of 49% of NPGs were female. Moreover, 40% of PGs and 32% of GHPGs were female. PGs and GHPGs deposited less money, bet less money, and gambled less frequently than NPGs. On average, GHPGs lost more money per gambling day (€122.15) as well more money as per session (€71.78) than all the PGs (€-68.24; €-42.73). On average, NPGs deposited money once (1.04) per session. PGs deposited 1.41 times per session and GHPGs deposited 1.53 times per session. On average, PGs (€63) and GHPGs (€96.08) deposited more money per session than NPGs (€49.35). Two-thirds of NPGs (65%) typically gambled until less than €5 was left in their gambling account. The respective values for PGs and GHPGs were 78% and 79%. A total of 12% NPGs had play breaks in the 30 days prior to answering the PGSI questions, 46% of PGs had play breaks and 59% of GHPGs had play breaks. The average money bet per game by PGs was €3.30, and GHPGs bet €5.73 per game. On average, NPGs bet €3.21 per game. The same pattern was found for the standard deviation of the bet. NPGs average standard deviation of the bet was €3.54, PGs average standard deviation of the bet was €3.79 and the GHPGs standard deviation of the bet was €6.79. The average profile of PGs and NPGs was similar to the findings in the cluster analysis. Compared to the entire group of PGs, GHPGs deviated more from NPGs with respect to all the metrics listed in Table 2.

**Table 2:** Average values for problem gamblers, greater harm problem gamblers, and non-problem gamblers

	PGs	GHPGs	NPGs
N	248 (26%)	79 (8.4%)	697 (64%)
Age	37	36	43
Female	40%	32%	49%
Amount deposited	432	478	631
Amount of money bet (€)	3253	2705	7032
Number of gambling days	5.79	4.76	8.30
Average monetary loss per gambling day (€)	-68.24	-122.15	4.22
Average number of deposits per session	1.41	1.53	1.04
Average amount of money deposited per session (€)	63.00	96.08	49.35
Average money loss per session (€)	-42.73	-71.87	-0.06
Percentage of sessions ending with low account balance	78%	79%	65%
Play break (yes/no)	46%	59%	12%
Average bet per game (€)	3.30	5.73	3.21
Standard deviation bet (€)	3.79	6.79	3.54

## Discussion

Between September 2021 and February 2022, 1,287 players of a European online gambling site answered the nine questions of the Problem Gambling Severity Index (PGSI). The frequency of the single PGSI scores ranging from 0 to 27 is displayed in Fig. 1. As expected, the distribution is skewed with more players scoring in the lower range and fewer players scoring in the higher range. However, there is a discrete step between scores of 26 and 27 on the PGSI. Sixty participants (4.66%) answered all nine questions of the PGSI with “almost always”. The present authors speculate that this spike was caused by participants who did not read the questions sufficiently and simply answered each question *“almost always”.* A similar spike was observed for players answering each question *“never”.* The authors also had access to the time from navigating to the PGSI page and pressing the submit button after answering all nine questions. After removing participants with unreasonably short response times, the spike with score of 27 disappeared (see Fig. 1). As far as the present authors are aware, this is the first time that a study has accurately measured the response times taken to complete a problem gambling screen. The results clearly indicate that response time can be a crucial aspect in improving data quality.

In the present study, 26% of participants were PGs, which corresponds to a PGSI score of 8 or above. The relatively high rate of self-reported problem gambling among the present sample of online casino players is in line with previous findings. Lopez-Gonzalez et al. (2018) collected responses to the PGSI in a sample of 659 Spanish sports-bettors. One-fifth of them had a score of 8 or above and were classed as PGs (19.1%). Håkansson and Widinghoff (2020) surveyed a sample of 1,004 Swedish online gamblers examining problem gambling symptoms (using the PGSI). They reported 44% of both past 30-day online casino gambling and live betting were problem gamblers. Moreover, 18% of those reporting online casino gambling but no live betting were problem gamblers.

The high percentage of problem gamblers in self-report studies is in stark contrast to previous studies classifying problem gamblers using pure behavioral tracking data. McAuliffe et al. (2022) reported that less than 1% of players were regarded as high-risk based on the Senet Group’s markers of harm. They also reported that *Entain*, which was part of the group of companies which defined the markers of harm, identified less than 6% of players as being high risk. Based on player tracking data, Catania and Griffiths (2021a) found that only 1.21% of their sample displayed elevated values on DSM-5 criteria for gambling disorder (although 33% were classed as at-risk gamblers). The large discrepancy between actual gambling expenditure and self-reported gambling identified by previous studies (Auer & Griffiths, 2017; Braverman et al., 2014) could play a role for the explanation for the discrepancy between the frequency of self-reported PG gambling and the proportion of high-risk players based behavioral tracking data.

Two AI algorithms were used to predict PG-based player tracking features 30 days prior to answering the PGSI. A random forest model achieved an AUC of 0.729 and a gradient boost machine model achieved an AUC of 0.67. Both goodness of fit statistics were computed on a test set which was left out from the model training. This level of model accuracy is in line with previous results. Louderback et al. (2021) used the Brief Biosocial Gambling Screen to assess self-reported problem gambling and they reported AUC values between 0.580 and 0.657. Luqiens et al. (2016) predicted self-reported problem gambling (using the PGSI) in a sample of online poker players and reported an AUC of 0.73.

In order to provide greater insights into the association between the player tracking features and self-reported problem gambling, a cluster analysis was performed. One cluster contained 43% PGs and players lost more money per gambling day and session, deposited more frequently per session, and depleted their gambling account in sessions more frequently. They also had more play breaks in the 30 days prior to answering the PGSI. However, in total they deposited less money, bet less money, and played less frequently. The higher likelihood of depositing within sessions and the higher likelihood of depleting the online account within-session could be indications of impaired self-control. Previous studies have suggested that online gambling might have negative impacts on self-control (Siemens et al., 2011). Two previous player tracking studies used frequent depositing as proxy measures for chasing losses. Perrot et al. (2018) operationalized chasing losses as either three or more deposits within a 12-hour period or a deposit less than one hour after a previous bet. One subgroup of players was characterized by a high gambling activity and a high probability of chasing behavior. Challet-Bouju et al. (2020) used the same operationalization of chasing losses as Perrot et al. (2018). In a cluster analysis they found a segment of players with a high gambling activity which was associated with a high number of chasing episodes.

The present authors developed a subgroup of PGs based on three PGSI items which appear to be more strongly associated with problem gambling (*“Have you felt that you might have a problem with gambling?”, “Has gambling caused you any health problems, including stress or anxiety?”* and *“Has your gambling caused any financial problems for you or your household?”*). A total of 8.4% of players answered at least one of these three questions with *“almost always”.* This subgroup of PGs (‘greater harm problem gamblers’ [GHPGs]) lost more money per session and per active gambling day and deposited money more frequently per session. In total they gambled and deposited less than all PGs. Three-fifths of the GHPGs (59%) had play breaks compared to 46% of all PGs. This is in line with the expectations as the GHPGs’ health and/or financials were impacted by gambling.

In the 30 days prior to answering the PGSI, PGs and GHPGs bet and deposited less than NPGs. However, the PGs and GHPGs deposited more per session, lost more money per session and day, and deposited more frequently per session. At first glance this seems contradictory. The explanation lies most likely in the fact that PGs were much more likely to self-exclude at some point of time during the 30 days prior to answering the PGSI. This limited the number of days on which they could gamble which reflects in the lower number of gambling days compared to NPGs. It is concluded that PGs played less frequently due to self-exclusion, but on the days they gambled, they spent more than NPGs. This is in line with previous studies which found that PGs spend more money than NPGs (Louderback et al., 2021; Luqiens et al., 2016). The increased likelihood of self-exclusions among PGs supports the notion that self-exclusion behavior is correlated with PG. Several previous studies have used self-exclusion as a proxy for problem gambling (e.g., Dragicevic et al., 2015; Percy et al., 2016; Finkenwirth et al., 2021).

One of the most influential metrics reported by the random forest algorithm was age. This was also evident in the cluster analysis and in the average profiles of PGs and NPGs. PGs were younger than NPGs. In their analysis of online poker players, Luqiens et al. (2016) also found PGs to be younger than NPGs. Although gender was not selected by the machine learning algorithms, there was a clear difference between PGs and NPGs. That difference was also evident in the cluster. The percentage of females in the PG group was lower compared to that in the NPG group. Previous studies have also found problem gambling to be more likely among males than females (e.g., Economou et al., 2019; Fröberg et al., 2015; Husky et al., 2015).

The most important variables predicting self-reported problem gambling were age, amount of money deposited, amount of money bet, number of gambling days, average monetary loss per gambling day, average monetary loss per session, average number of monetary deposits per session, account depletion, and number of play breaks. However, analysis of these variables does not provide information about the direction of the association between independent variables and the dependent variable. Younger players for example might have an elevated risk or a decreased risk. Consequently, additional cluster analysis was performed which provided additional evidence concerning the variables most predictive of problem gambling.

The findings regarding frequent depositing and depleting the gambling account balance are particularly interesting because they are fundamental to most online gambling operators’ marketing practices. Players have to deposit before they can play and to the best of the present authors’ knowledge online gambling operators are trying to make this process as easy and as frictionless as possible. Often players can deposit with one click and/or are reminded when their account balance decreases. The present study’s findings question these practices and suggest that frequent depositing should be made more difficult. The present authors are not aware of any regulation which would limit depositing frequency in short time periods or prohibit operators from enticing monetary depositing within sessions.

The findings will be of interest to many different stakeholder groups including the gambling industry, gambling policymakers, gambling regulators and researchers in the gambling studies field. The findings provide empirical evidence concerning the most important behavioral indicators of problem gambling which could be used by (i) the gambling industry to help identify problem gamblers using account-based data, (ii) gambling policymakers and regulators to make evidence-based informed decisions and policies in the area of player protection and harm-minimization, and (iii) researchers in the gambling studies field to replicate and/or build on the findings reported here with other samples from different gambling operators and different countries.

### Limitations

The present study has a number of limitations that should be considered when interpreting the study’s key findings. First, only a relatively small number of participants answered the PGSI questions which was then used to train the AI algorithms. Second, the PGSI data were self-report and therefore subject to established methods biases (e.g., social desirability). However, given that the self-report data appeared to support the objective player tracking data, the self-report data would appear to have good face validity. Third, the study was conducted with players from just one European online gambling operator during a specific (and relatively short) period of time and therefore the data are not necessarily representative of online gamblers more generally. The results could vary across operators and jurisdictions, as well as other time periods. However, the main findings are in line with the findings from previous research which identified that frequent session deposits were correlated with higher gambling intensity. Additionally, the study’s validity was further improved by the fact that the response time to the PGSI was measured and used to help identify potentially unreliable answers. As this is the first study to correlate self-reported problem gambling with player tracking data, future replication studies should be conducted with data from different operators in other jurisdictions and utilize larger sample sizes and study the gambling behavior for longer time periods (e.g., six months or a year).

## Conclusions

The present study showed that self-reported problem gambling can be predicted by AI algorithms with high accuracy based on player tracking data. The reported model accuracies were in line with previous prediction studies in the area of responsible gambling. The results also supported Auer and Griffiths’ (2016) assertion that not all PGs self-exclude and vice versa. The GHPGs spent more money, deposited money more frequently within sessions, and depleted the gambling account more frequently compared to all PGs and NPGs. However, numerous jurisdictions require operators to identify problem gambling based on behavioral tracking data. For example, Sweden requires operators to monitor younger players more thoroughly (Svenska Spel, 2020). This is supported by the fact that PGs were younger in the present study. The findings of the present study shed more insight into significant metrics and demographic differences concerning problem gamblers by using a mix of objective (account-based tracking) data and subjective (self-report) data.

## Data Availability

The data for this study are commercially sensitive and are not publicly available.

## Appendix 1: Problem Gambling Severity Index items

**Table:**

Item number and question
(1) Have you bet more than you could really afford to lose?
(2) Have you needed to gamble with larger amounts of money to get the same excitement?
(3) Have you gone back to try to win to back the money you’d lost?
(4) Have you borrowed money or sold anything to get money to gamble?
(5) Have you felt that you might have a problem with gambling?
(6) Have you felt that gambling has caused you any health problems, including stress or anxiety
(7) Have people criticized your betting, or told you that you have a gambling problem, whether or not you thought it is true?
(8) Have you felt your gambling has caused financial problems for you or your household?
(9) Have you felt guilty about the way you gamble or what happens when you gamble?

Individuals can answer: Never (0), Sometimes (1), Most of the Time (2), Almost Always (3)

## Appendix 2: Player tracking features based on the 30 days prior to answering the PGSI

**Table:**

Feature Number	Feature
1	Age (in years)
2	Gender
3	Number of play breaks
4	Number of voluntary limit changes
5	Number of bets
6	Amount of money bet
7	Average bet amount
8	Standard deviation bet
9	Number of deposits
10	Amount of money deposited
11	Standard deviation deposits
12	Amount of money won
13	Amount of money lost (amount won minus amount bet)
14	Number of sessions
15	Total session length (in minutes)
16	Number of different gambling days
17	Average number of deposits per gambling day
18	Average number of deposits per session
19	Average amount of money lost per gambling day
20	Average monetary loss per session
21	Average amount of money deposited per gambling day
22	Average amount of money deposited per session
23	Percent of sessions ending with low account balance
